# Qualitative and quantitative aspects of the F-A-S fluency test in people with aphasia

**DOI:** 10.1590/1980-57642020dn14-040012

**Published:** 2020-12

**Authors:** Isabella Linnea Jansson, Karin Zazo Ortiz, Simone dos Santos Barreto

**Affiliations:** 1 Department of Speech-Language Pathology, Universidade Federal Fluminense – Nova Friburgo, RJ, Brazil.; 2 Department of Speech Language and Hearing Sciences, Universidade Federal de São Paulo – São Paulo, SP, Brazil.; 3 Department of Specific Training in Speech-Language Pathology, Nova Friburgo Health Institute, Universidade Federal Fluminense – Nova Friburgo, RJ, Brazil.

**Keywords:** language tests, neuropsychological tests, cognition, language, aphasia, testes de linguagem, testes neuropsicológicos, cognição, idioma, afasia

## Abstract

Verbal fluency tests are widely used in neuropsychological assessment. The quantitative and qualitative analysis of the performance of aphasic individuals on the phonemic verbal fluency (PVF) test can contribute to a better understanding of cognitive changes in this group of patients.

**Objective:**

This study aimed to analyze clustering and switching measures of PVF in people with aphasia and investigate the relationship between the use of these strategies, the quantitative performance on the test, and the performance on executive functions.

**Methods:**

This is a cross-sectional study of 15 people with aphasia, right-handed, native speakers of Brazilian Portuguese, aged 19 to 92 years. They were submitted to the F-A-S test and the Clock Drawing Test (CDT). The following measures were obtained in the F-A-S test: total score, number of clusters, mean cluster size, and number of switches. Spearman's test was used to analyze correlation.

**Results:**

We found a positive correlation among all F-A-S test scores, ranging from p=0.61 (p<0.001) to p=0.91 (p<0.001). No correlation was identified between these measures and CDT performance (p≤0.31; p≥0.260).

**Conclusions:**

The quantitative and qualitative analysis of F-A-S in people with aphasia, even those with different linguistic manifestations, showed that these individuals presented lower scores and that the number of total words and the number of switches were strongly correlated. We found no correlation between executive function, assessed by the CDT, and switching performance on the F-A-S test.

## RESUMO.

Testes de fluência verbal são procedimentos amplamente utilizados na avaliação neuropsicológica. A análise da performance de pessoas com afasia no teste de fluência verbal fonêmica (FVF), do ponto de vista quantitativo e qualitativo, pode contribuir para uma melhor compreensão das mudanças cognitivas que ocorrem nesse grupo de pacientes.

**Objetivo:**

Os objetivos deste estudo foram analisar as medidas de estratégias de agrupamento e de mudança de agrupamento na FVF de pessoas com afasia e investigar a correlação entre o uso dessas estratégias, o desempenho quantitativo no teste e o desempenho em funções executivas. Métodos: Foi realizado um estudo transversal com 15 pessoas com afasia, destros, falantes nativos do português brasileiro, com idade entre 19 e 92 anos. Eles foram submetidos ao teste F-A-S e ao Teste do Relógio (TDR). Foram obtidas as seguintes medidas no F-A-S: escore total, número e tamanho médio de agrupamento e número de mudança de agrupamento. Para análise de correlação foi utilizado o teste de *Spearman*.

**Resultados:**

Foi verificada correlação positiva entre todos os escores do teste F-A-S, variando entre p=0,61 (p<0,001) e p=0,91 (p<0,001). Não foram encontradas correlações entre tais medidas e o escore no TDR (p≤0,31; p≥0,260).

**Conclusões:**

A análise quantitativa e qualitativa do F-A-S de pessoas com afasia, mesmo com diferentes manifestações linguísticas, mostrou que elas apresentam escores inferiores e o número total de palavras e o número de mudanças de agrupamento possuem forte correlação. Não foi observada correlação entre funções executivas, avaliada pelo TDR, e o desempenho na medida de mudança de agrupamento.

## INTRODUCTION

Verbal fluency tests (VFTs) are widely used in neuropsychological assessments to identify cognitive disorders due to neurological injuries, such as dementia, traumatic brain injury, stroke, and tumor, and other neurological or psychiatric conditions.^1^ ^,^ ^2^ Although VFTs are very useful in identifying lexical difficulties, other important cognitive aspects can be analyzed during its application. Firstly, some authors conceptualized VFTs as instruments to measure executive function.^3^ Then, other researchers assumed that VFTs were only loaded onto the language factor.^4^ Although VFTs are strongly associated with lexical ability and access, lexical-semantic network, word and semantic knowledge, and auditory attention, they are also related to multiple aspects of executive functions.^5^

The three forms of VFTs assessed are phonemic, verbal, and semantic. In these tests, participants are asked to provide responses in a limited time frame (usually one minute) according to the form being used.^2^ ^,^ ^6^ ^,^ ^7^ In the semantic fluency test, the individuals have to produce as many words as possible belonging to a certain semantic category predefined by the examiner (e.g., animals). In the fluency task with verbal criteria, only verbs can be produced. Finally, in orthographic or phonemic verbal fluency (PVF), the participants have to produce words beginning with a specific letter or phoneme.^2^ ^,^ ^6^ ^,^ ^8^

The PVF F-A-S test is one of the most used instruments to assess people with neurological disorders.^2^ ^,^ ^6^ ^,^ ^8^ ^–^ ^10^ They can be found in screening procedures or full language assessment batteries, including those for aphasia. Aphasia is caused by focal damage to cortical and/or subcortical structures of the dominant hemisphere(s) for verbal symbolic manipulations. It affects and is affected by other physiological information processes to the degree that they support, interact with, or are supported by symbolic deficits.^11^ Thus, VFTs are used for language assessment of people with aphasia (PWA).

Beyond the controversies about what exactly PVFs evaluate (language and/or executive functions), one point worth mentioning is the importance of analyzing strategies used during the phonological search. The task strategies are clustering and switching.^12^ ^–^ ^14^ Clustering is the production of groups of words according to meaning or sound. It depends on verbal memory processes related to the temporal lobe. When a given category ends, a switching occurs, which demands mental flexibility and is more closely related to the frontal lobe.^12^ ^–^ ^14^

The F-A-S test also allows the quantitative assessment of performance based on the total score (number of valid words produced), which is the most common use.^2^ ^,^ ^8^ ^,^ ^9^ Despite being a widely used procedure, few studies have investigated the performance of PWA on the PVF task specifically regarding the use of cognitive strategies.^4^ ^,^ ^5^ ^,^ ^15^ Recent investigations analyzing the use of these strategies in PVF tests did not involve PWA^4^ ^,^ ^5^ or used diagnostic criteria that did not clearly differentiate aphasic from non-aphasic groups.^15^ The quantitative and qualitative analysis of the performance of aphasic individuals on the PVF test can contribute to a better understanding of cognitive changes in this group of patients. Besides, the PVF performance of PWA is possibly different and may not be extended to other populations referred to clinical neuropsychological assessments. One of the hypotheses of the present study is that the use of clustering and switching strategies might be associated with the number of words generated in the PVF F-A-S test by PWA.

How language and other cognitive aspects, such as attention and executive functions, interact^16^ during this test is another issue that still needs clarification. Therefore, we also investigated whether the qualitative analysis (based on clustering and switching strategies) is related to results from a non-verbal test of executive functions, that is, if PVF can really give us information about changes in cognitive domains other than language.

This study aimed to analyze clustering and switching measures of PVF in PWA and investigate the relationship between the use of these strategies, the quantitative performance on the test, and the performance on executive functions.

## METHOD

This is an observational cross-sectional multicenter study. The study was approved by the Ethics Committee of both institutions (decisions no. 2,562,263 and 2,658,992). All patients signed informed consent forms prior to participation.

### Participants

The participants were recruited from two speech therapy outpatient clinics of two Brazilian universities. The general inclusion criteria were: no previous history of neurological or psychiatric disorder, alcoholism, or drug use; no use of psychotropic medications, except for atypical neuroleptics; and absence of visual or auditory impairments as well as severe auditory comprehension disorder, as they could affect the test outcome. This study excluded patients with scores below the cut-off point for their schooling and age on the word and sentence oral comprehension subtests of the Brazilian version of the Montreal Toulouse Language Assessment Battery (MTL-BR).^17^ ^,^ ^18^ Monolingual adults and older people, native speakers of Brazilian Portuguese (BP), with a speech-language diagnosis of aphasia were included in this study. All patients were right-handed, had been evaluated by a neurologist, underwent neuroimaging tests, and were assessed with the MTL-BR for aphasia.

Fifteen patients were selected for this study. The group was very heterogeneous as to age (19 to 92 years old) and schooling (from 3 to 12 years of formal education). The prevalent etiology was stroke, and most PWA were at the chronic stage (from 2 to 115 months). Table 1 presents sociodemographic and clinical data.

**Table 1:** Sociodemographic and clinical data from the sample.

Participants	Gender	Age	Educational level (years)	Duration (months)	Etiology	Brain injury	Type of aphasia
P01	M	92	5	12	IS	Unspecified	Wernicke's aphasia
P02	M	47	10	29	IS, TBI	Fronto-parieto-temporal (L)	TSA
P03	M	54	6	4	IS	Cortico-subcortical frontal (L)	Broca's aphasia
P04	M	60	11	4	HS	Frontal (L)	Mixed aphasia
P05	F	50	5	3	IS	Parietal (L)	Mixed aphasia
P06	F	61	5	2	IS	Frontoparietal (L)	Mixed aphasia
P07	F	46	3	7	IS	Cortico-subcortical parietal (R)	Anomic aphasia
P08	M	74	8	32	IS	Temporal (L)	Mixed aphasia
P09	M	19	10	65	IS	Frontotemporal (L)	Anomic aphasia
P10	F	49	8	115	IS	Frontotemporal (L)	Mixed aphasia
P11	F	72	8	47	IS	Frontoparietal (L)	Mixed aphasia
P12	M	29	12	77	TBI	Fronto-parieto-temporal (L)	Mixed aphasia
P13	M	72	11	25	IS	Frontotemporal (L)	Conduction aphasia
P14	F	55	11	8	IS	Cortico-subcortical fronto-parietal (L)	Anomic aphasia
P15	F	29	6	4	IS	Frontotemporal (L)	Broca's aphasia
**Mean**	-	53.9	7.9	28.9	-	-	-
**SD**	-	19.3	2.8	33.6	-	-	-

M: male; F: female; IS: ischemic stroke; HS: hemorrhagic stroke; TBI: traumatic brain injury; L: left; R: right; TSA: transcortical sensory aphasia; SD: standard deviation.

### Materials and Procedures

The F-A-S test was administered to assess PVF since it is the most studied version and enables a qualitative analysis of the speaker's performance.^2^ ^,^ ^6^ ^,^ ^9^ For the performance on the F-A-S test, the participants were asked to say as many words as possible, beginning with letters “F”, “A”, and “S” within a 1-minute period for each letter. The participants were instructed not to repeat items, say proper nouns or words derived from nominal or verbal inflections. An example with the letter “P” was provided by the examiner before the task. The letter P was selected due to its high frequency at the beginning of words in BP, similar to the incidence of letters F-A-S.^8^ The test complied with the criteria proposed by Senhorini et al.^6^ The procedure was recorded in audio format with a Moto Z Play 32GB mobile phone, model XT1635-02.

Answers were transcribed and analyzed according to the criteria proposed by Troyer et al.^12^ ^,^ ^13^ Four scores were obtained: number of valid words (errors and repetitions were excluded), number of clusters, mean cluster size, and number of switches. All measures were calculated based on the total words retrieved in the three letters. Errors were computed in clustering and switching measures, since they also allow assessing the cognitive strategies used by the participants during their performance on the task. In this study, we also analyzed the number of clusters.

A clustering was considered when the participant produced: successive words generated with the same two initial letters (alliteration), word endings (rhymes) and/or words that differed only by a vowel, and homonyms (words with the same spelling but different meanings), as long as the participant was aware of the difference between them. A cluster was counted when it had at least two words. In cases of overlay of two subcategories with items belonging to both, with some belonging exclusively to one and others belonging exclusively to the other, the overlaid items were considered in both categories. However, whenever a small clustering was part of a bigger one, only the larger subcategory was considered. Cluster size was counted from the second word, and single words had a zero score in the calculation of mean cluster size. Switching, which corresponds to the number of transitions between the clustering, was counted even when isolated words were produced.^12^

Besides clustering and switching measures, the type and frequency of other invalid words were also analyzed. Invalid words were classified into the following categories: intrusion (production initiated by different phoneme), perseveration (repetition of previous production, except valid words that were computed as repetition, according to the analysis proposed by Troyer et al.^12^ ^,^ ^13^), and neologism.

The Clock Drawing Test (CDT) investigated executive functions,^19^ ^–^ ^21^ which are also important in the performance of the F-A-S test. A non-verbal test was chosen due to the lack of validated verbal tests for executive function assessment in PWA, given their linguistic impairment. In addition, evidence shows that PWA perform worse on this test when compared to non-aphasic brain-injured patients.^22^ The cut-off point used for the study was 6.^23^ ^–^ ^25^ In addition to the CDT, the Geriatric Depression Scale (GDS) was administered^26^ The GDS was not adopted as an exclusion criterion because most PWA have depressive symptoms. Risk of depression was identified in 60% of the sample. However, subgroups with and without risk of depression were compared, and no differences were found between them on the F-A-S test measures.

Data were expressed as means of measures of central tendency and dispersion and relative frequency. For data analysis, we verified the non-adherence of numerical variables to the normal curve, using the Shapiro-Wilk test. The Spearman's test was applied to analyze the correlation among the F-A-S test scores and the CDT performance. Correlation strength was analyzed according to Siegel's^27^ proposal. The significance level adopted was p<0.05.

## RESULTS

The mean PVF F-A-S test score of PWA was 5.9 words, with a standard deviation (SD) of 4.9. With respect to the cognitive strategies used by the participants, the mean scores for clustering (number and mean size) and switching were, respectively, 1.3 (SD=1.1), 0.9 (SD=2.5), and 1.9 (SD=2.3). Table 2 shows the results for each participant.

**Table 2:** Performance in the F-A-S test and clock drawing test.

Participants	Total score (F-A-S)	N-CL (F-A-S)	M-CLS (F-A-S)	N-SW (F-A-S)	CDT
P01	1	0	0	0	4
P02	5	1	1	0	3
P03	2	1	0	0	10
P04	4	1	0.3	1	1
P05	18	4	9.8	6	1
P06	4	1	0.5	1	2
P07	11	3	0.3	5	4
P08	2	1	0.3	0	5
P09	11	1	0.2	6	4
P10	5	1	0.3	1	9
P11	0	0	0	0	3
P12	1	0	0	0	4
P13	8	2	0.3	3	10
P14	9	2	0.3	4	4
P15	8	1	0.5	1	9
**Median**	5.0	1.0	0.3	1.0	4.0
**Mean**	5.9	1.3	0.9	1.9	4.9
**Minimum**	0	0	0	0	1
**Maximum**	18	4	9.8	6	10
**SD**	4.9	1.1	2.5	2.3	3.1

Legend: N-CL: number of clusters; M-CLS: mean cluster size; N-SW: number of switches; CDT: clock drawing test.

Only four participants produced errors, according to the Troyer et al.^12^ criteria: four proper names, one derived word, and two repetitions. Eight participants generated 100 invalid words, of which 45% corresponded to intrusions, 28% to perseveration, and 27% to neologisms.

Table 3 presents the correlation among total score, clustering (number and mean size), and switching (number) measures in the F-A-S test. The total F-A-S test score showed a strong to moderate positive correlation with the clustering and switching measures analyzed.

**Table 3:** Correlations among the scores for number of clusters, mean cluster size, and number of switches and the total scores for the F-A-S test and clock drawing test.

	Total F-A-S score	CDT score
Number of clusters	0.86 (<0.001*)	0.01 (0.966)
Mean cluster size	0.61 (<0.001*)	-0.31 (0.260)
Number of switches	0.91 (<0.001*)	-0.13 (0.633)

* Spearman's rank correlation coefficient; (p)<0.05; CDT: clock drawing test.

Regarding the CDT test, which assessed the performance of executive functions, the mean score was 4.9 (SD=3.1). Table 3 also presents the correlation between the F-A-S test and CDT measures. We found no correlation between the total score or the use of clustering and switching strategies and the CDT performance.

## DISCUSSION

PWA performed poorly on the PVF test, both on the number of words retrieved and the cognitive strategies used to complete the task. Firstly, the mean number of words generated by the participants was considerably lower than the mean scores of previous studies carried out with healthy adult and older Brazilians (25.4 to 43.5).^2^ ^,^ ^9^ ^,^ ^28^ This result can be attributed to brain injury in the left hemisphere^15^ but mainly to the aphasic condition.^22^ In addition, the low educational level of the participants could also have interfered with the results (all participants, except one, had less than 11 years of formal education), as observed in a study of post-stroke patients with low schooling.^15^

The mean total number of words found in this study was similar to that of a previous study that investigated the performance of PWA on the F-A-S test, in which the mean and SD were 3.2 and 4.5.^22^ The reduced number of words generated in the PVF test by PWA might be associated with impaired access to the phonological lexicon, as processing occurs in the left brain hemisphere,^15^ often damaged in these patients.

Regarding the use of clustering and switching strategies, mean scores for number of switches were also lower when compared to previous studies of healthy subjects.^12^ ^,^ ^13^ These studies presented the following scores: mean cluster size ranging from 0.4 to 0.5^12^ ^,^ ^13^ and number of switches, from 27.0 to 29.6.^12^ ^,^ ^13^ Studies analyzing the number of clusters in the F-A-S test of adults were not found. The worst performance of PWA on number of switches suggests that, in addition to linguistic impairment (access to the phonological lexicon), the use of cognitive flexibility to search for new strategies to perform the task might also be compromised.

The clustering and switching strategies used by PWA had the same pattern observed in studies with PVF tests in this population,^15^ especially for switching measures. Evidence shows that right hemisphere post-stroke patients did not present this pattern in PVF tests. In these cases, the number of switches was comparable to that of neurologically healthy people.^15^ This evidence reinforces the hypothesis that the reduced number of switches produced by PWA might be mainly associated with deficits in the specific linguistic processing that occurs in the left hemisphere since deficits in executive functions are not exclusive to this hemisphere.

The present study participants produced more invalid words than errors or repetitions considering the criteria proposed by Troyer et al.^12^ Regarding the invalid words, intrusions were the most common, followed by perseverations and neologisms. The occurrence of intrusions and perseverations could be related to inhibitory control failures. Although neologisms are a linguistic manifestation of the aphasic condition, their occurrence suggests phonological disintegration. Future analysis including other frequent types of language errors made by PWA can contribute to understanding the cognitive strategies used by them during the VFT.

Despite the lower PVF scores identified in PWA, strong to moderate positive correlations were found among the scores for number of clusters, mean cluster size, and number of switches and total F-A-S test score, as shown in Table 3. The total score in the test shows a strong correlation with the number of switches, as observed in healthy people.^12^ In PVF tasks, switching was the most used strategy by people without left hemisphere brain damage.^15^ According to Shao et al.,^29^ associations with words with the same initial letter are less frequent, so their access is more difficult. Namely, the reason for varying switching strategies is more effective in a PVF task. In the present study, the strong positive correlation found between the number of switches and the total number of words generated indicates that PWA use this cognitive strategy less often, leading to a smaller number of retrieved words.

Considering that switching is associated with mental flexibility, we expected to find a relationship between switching and CDT. However, such correlation was not detected (Table 3). This result reinforces that the use of cognitive strategies in the F-A-S test (including the number of switches) may not involve the executive functions assessed by the CDT. In fact, the debate about the relationship between language and other cognitive domains is not new. More recently, some authors have argued that the cognition involved in language processing is domain specific,^11^ that is, each cognitive domain is allocated in ways that are particular to specific tasks rather than as a general resource equivalently allocated to all processing tasks.^16^ This hypothesis suggests that choosing a non-verbal test for this purpose would not be appropriate.

Regarding the possible association between the use of clustering and switching and the integrity of the frontal and temporal lobes, respectively, the reduced sample and the heterogeneity of the brain lesions limited a specific analysis of this aspect. Five patients had a frontal lobe lesion, associated or not with a lesion in another brain area except the left temporal lobe (P03, P04, P06, P11, P14), and only one patient presented a lesion in the left temporal lobe without damage to the frontal lobe (P08). Scores for the number of clusters and mean cluster size from P08 did not differ from means of these measures in the subgroup with damage to the frontal lobe, and the number of switches was lower than the mean observed in these subgroups (0 *versus* 1.2). Therefore, we found no trend toward worse switching performance in PWA with injuries involving the frontal lobe, as observed by Troyer et al.^13^

This study evaluated a small number of PWA with different clinical conditions and sociodemographic characteristics. These aspects and the lack of other clinical groups or normal subjects limited our data interpretation. Nevertheless, the patients presented different language disorders (different types of aphasia) and were assessed by a standardized language battery specific to this population.

The quantitative and qualitative analysis of F-A-S in PWA, even those with different linguistic manifestations, showed that these individuals presented lower scores and that the total number of words and the number of switches were strongly correlated. The present study found no correlation between executive functions, assessed by the CDT, and switching performance on the F-A-S test. Such results suggest that the PVF performance by PWA could not be explained only by an executive impairment, but mainly by the linguistic deficit. The switching strategy analysis, coupled with the analysis of other errors commonly found in PWA, can contribute to a better understanding of the phonological-lexical access difficulties in this population, as well as the use of other cognitive strategies to perform the task, with possible implications for the rehabilitation process.
